# Self-motion direction estimation from optic flow is a result of capacity-free and implicit ensemble coding

**DOI:** 10.1177/20416695251377199

**Published:** 2025-09-15

**Authors:** Qian Sun, Haojiang Ying, Qi Sun

**Affiliations:** 1 66344 School of Psychology, Zhejiang Normal University, Jinhua, China; 2 Intelligent Laboratory of Zhejiang Province in Mental Health and Crisis Intervention for Children and Adolescents, Jinhua, China; 3 Key Laboratory of Intelligent Education Technology and Application of Zhejiang Province, Zhejiang Normal University, Jinhua, China; 4 Department of Psychology, 12582 Soochow University, Suzhou, China

**Keywords:** heading perception, ensemble perception, optic flow, automatic

## Abstract

Numerous studies have explored the mechanisms of heading estimation from optic flow and ensemble coding in other features, yet none have examined ensemble coding's role in heading estimation. This study addressed this gap through two experiments. Participants sequentially viewed three (experiment 1) or five/seven (experiment 2) optic flow-simulated headings, then reported specific directions. Results revealed that individual heading accuracy declined with increasing numbers, while estimates closely matched ensemble representations, demonstrating ensemble coding in heading estimation. Notably, ensemble coding accuracy remained unaffected by heading quantity, indicating its capacity-free nature—unlike capacity-limited individual heading processing. The discovered summary statistics of motion may help us to better understand the navigation in complex environments (e.g., how pedestrians/drivers judge their self-motion directions), which could potentially contribute to real-world implications.

## How to cite this article

Sun, Q., Ying, H., & Sun, Q. (2025). Self-motion direction estimate from optic flow is a result of capacity-free and implicit ensemble coding. *i-Perception, 16*(5), 1-12. https://doi.org/10.1177/20416695251377199

## Introduction

To accurately estimate our self-motion direction (i.e., heading), our visual and cognitive systems use various visual and nonvisual information (Angelaki et al., 2009; Chen et al., 2013; Fetsch et al., 2010; Schindler & Bartels, 2018). Among them, it has been demonstrated that observers can accurately estimate their translational heading directions from optic flow (Gibson, 1950)—a dynamic light-motion pattern projected on an observer's retina when one is moving in the world (Burlingham & Heeger, 2020; Crowell & Banks, 1993; Layton & Fajen, 2016; Maus & Layton, 2022; Sun et al., 2024a, 2024b; Warren & Hannon, 1988; Warren et al., 1988) and systematically show a bias toward the straight-ahead direction, which is known as center bias (e.g., D’Avossa & Kersten, 1996; Sun et al., 2024a, 2024b; Xu et al., 2022). Additionally, recent studies have demonstrated that heading perception from optic flow also involves different cognitive abilities, such as attention (Sun et al., 2024b) and working memory (Sun et al., 2023).

**Figure 1.**
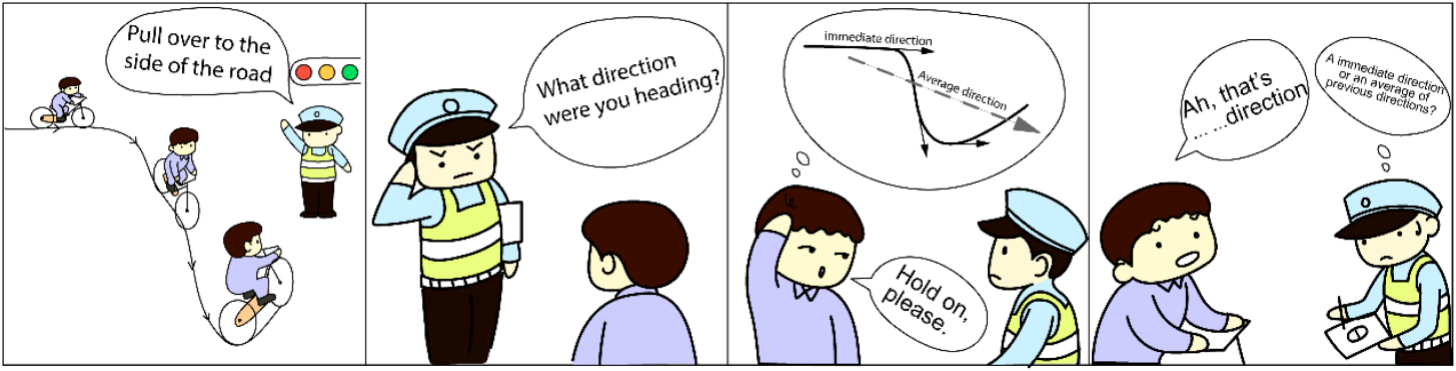
Schematic depiction of a traffic violation interaction between law enforcement and a cyclist.

However, the above studies on heading perception present only one optic flow pattern per trial, requiring participants to report a single heading direction. This approach examines memory for isolated events, unlike real-world scenarios. For example, when a traffic officer asks “Which direction were you heading?”, we may report either our immediate direction or an average of recent headings—a distinction current literature fails to address (Figure 1).

Capacity-limited working memory (Oberauer et al., 2016) and efficient coding principles (Attneave, 1954) suggest that remembering individual headings is cognitively costly. Instead, observers likely compute an average—consistent with *ensemble coding*, where people efficiently extract summary statistics from multiple items (Whitney & Leib, 2018). This effect generalizes across features like orientation (Utochkin et al., 2024), motion direction (Sweeny et al., 2013), and facial traits (Haberman & Whitney, 2009). Moreover, such phenomenon does not only occur at perception of simultaneously presented stimuli, but also at sequentially presented ones (e.g., Haberman et al., 2009; Ying et al., 2020). Notably, Sweeny et al. (2013) demonstrated ensemble coding for *object* motion, raising the question: does it also apply to *self-motion direction* (heading) estimation?

Ensemble coding is often considered a capacity-free process that bypasses visual system limitations (Alvarez, 2011; Epstein & Emmanouil, 2017; Fitousi, 2025). For example, mean size estimation remains accurate regardless of set size (Attarha et al., 2014), though basic attentional resources are still required (Alvarez & Franconeri, 2007; Huang, 2015). However, representing multiple ensembles may be a limited-capacity process (Fitousi, 2025) and result in degraded individual item precision (Haberman & Whitney, 2007). This raises critical questions for heading estimation: While optic flow integration relies on component motion trajectories (Warren et al., 1988), recent work shows attention and working memory constrain individual heading judgments (Sun et al., 2023, 2024a, 2024b). If ensemble coding operates in multiheading contexts, does it remain capacity-free? And is it truly independent of individual heading processing?

In summary, the current study investigated whether heading estimation from optic flow involves capacity-free ensemble coding through two experiments adapting Khayat & Hochstein's (2018) paradigm. Participants sequentially viewed three to seven optic flow patterns before reporting specific *n*th headings, with results showing three key findings: (1) serial position effects (enhanced accuracy for first/last headings), (2) systematic bias toward the mean heading direction indicating ensemble coding, and (3) invariant ensemble accuracy across set sizes, demonstrating its capacity-free nature. These findings establish that heading estimation automatically integrates multiple flows into summary representations independent of working memory constraints, revealing a fundamental mechanism for efficient navigation in complex environments.

## Experiment 1

### Methods

#### Participants

Eighteen participants (11 females, seven males; 19–25 years old) were enrolled from Zhejiang Normal University. All were naïve to the experimental purpose and with normal or corrected-to-normal vision. The sample size was determined according to the previous studies (e.g., Warren et al., 1988; Sun et al., 2024a, 2024b). The Scientific and Ethical Review Committee in the School of Psychology of Zhejiang Normal University approved the experiment.

#### Stimuli and Apparatus

**Figure 2.**
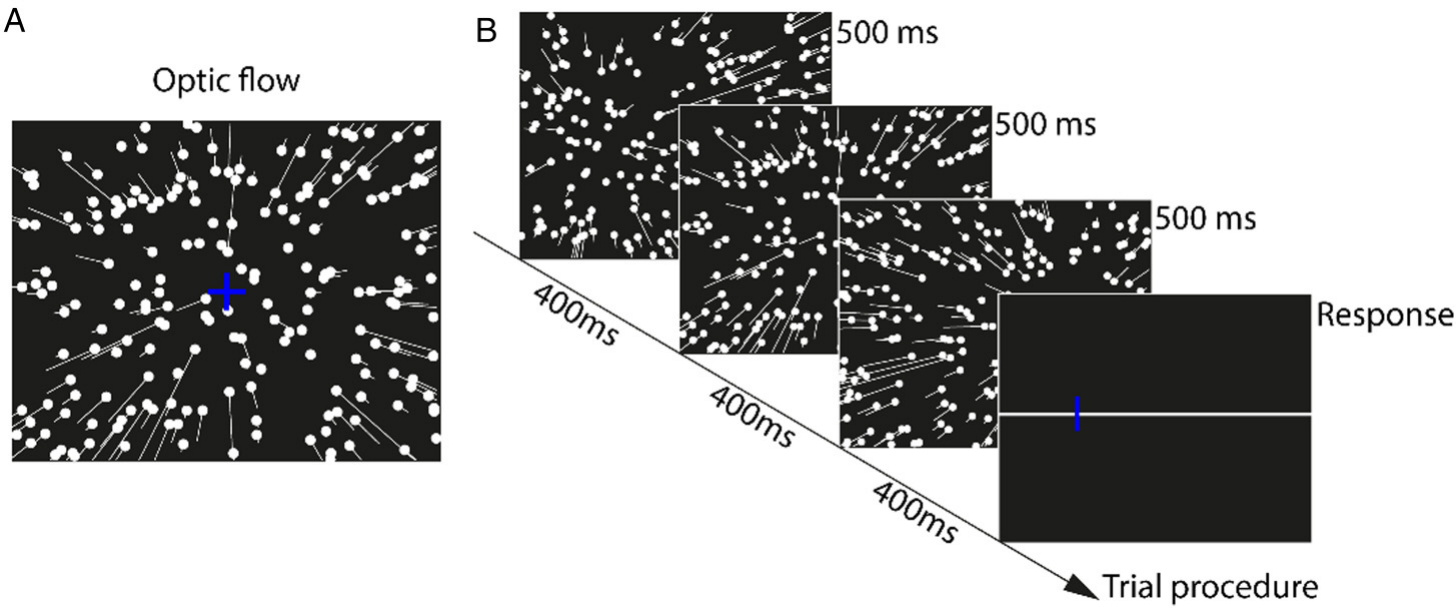
(A) Schematic representation of experimental visual stimuli simulating observer translation through a 3D dot-cloud. Dots represent initial positions (frame 1), while white lines (not visible during experiments) indicate subsequent motion trajectories. (B) Illustration of a trial produce consisting of three optic flow displays.

The current study presented three sequential optic flow patterns (Figure 2A) per trial (112°H × 80°V), simulating observer translation through a three-dimensional (3D) dot-cloud (200 dots, 0.28° diameter, 22.5 cd/cm^2^ luminance) at 1.5 m/s speed with depth ranging from 0.2–5 m. Each flow pattern's heading direction was randomly selected from seven possible angles (0°, ± 10°, ± 20°, ± 30°), where negative/positive values indicated left/right deviations from screen center (0°).

The visual stimuli were generated using MATLAB (Psychophysics Toolbox 3) and displayed on a 27-inch Dell monitor (2560 × 1440 resolution, 59.8 × 33.6 cm, 60 Hz refresh rate) driven by an NVIDIA GeForce GTX 1660Ti graphics card.

#### Procedure

Participants were seated in a light-exclude room with their heads stabilized using a chin-rest, maintaining strict head–body alignment with the display center. They viewed the stimuli monocularly (right eye) at a fixed 20cm distance to minimize binocular disparity conflicts while preserving simulated motion parallax cues. Throughout the experiment, participants maintained central fixation and refrained from any head or body movements.

As shown in Figure 2B, each trial consisted of three sequentially presented 500ms optic flow patterns, each followed by a 400ms blank interval. Following the final blank, a central cue number (n = 1, 2, or 3) indicated which of the three headings participants should recall. Simultaneously, a 112° horizontal line appeared with a randomly positioned blue vertical bar, which participants adjusted to match the cued heading direction before confirming their response via mouse click.

Heading directions were randomly selected from seven possible angles (0°, ± 10°, ± 20°, ± 30°) across 270 experimental trials. Prior to testing, participants completed 10 practice trials (excluded from analysis) to familiarize themselves with the procedure. The entire session lasted approximately 20 min.

#### Data Analysis

We recorded the heading estimate of each trial. To examine whether participants could accurately retrieve and discriminate the target heading directions, we fitted the heading estimates ( $HE_{i}$ ) as a linear function of their corresponding target heading direction ( $TH_{i}$ ), given by:

(1) $$HE_{i}=s_{i}\times TH_{i}$$

where *i* indicates the *i*th presented target heading directions (*i* = 1, 2, 3, etc.). Additionally, we also fitted the heading estimates of all trials as a linear function of the previous *j*th (*j* = 1, 2, *i-1*) heading directions, given by:

(2) $$HE=s_{j}^{\prime }\times TH$$

If participants could remember and discriminate the target heading direction, then $s_{i}$ would be significantly larger than $s_{j}^{\prime }$ .

Moreover, given that previous studies have demonstrated that the heading estimates are systematically compressed toward the straight-ahead direction (0°), indicating a center bias (e.g., Sun et al., 2023, 2024a, 2024b), it can be expected that $s_{i}$ will be significantly smaller than 1 ( $s_{i}$ = 1, indicating that the heading estimate equals to the actual heading). Accordingly, the larger the $s_{i}$ is, the more accurate the estimation is.

Aside from the question above, we also examined whether participants represented the presented headings by ensemble encoding/averaging (i.e., ensembled heading). If true, what weights were assigned to the different headings? To address these questions, we conducted two types of multifactors linear regression, given by:

(3.1) $$HE=w_{0}\times \frac{1}{n}\left(\overset{n}{\underset{i=1}{\sum }}\;w_{i}TH_{i}\right)$$

(3.2) $$HE=w_{0}^{\prime }\times \overset{n}{\underset{i=1}{\sum }}\frac{w_{i}}{\sum_{i=1}^{n}\;w_{i}}TH_{i}$$

In equations (3.1) and (3.2), we assumed that participants first create an ensemble heading by assigning weights ( $w_{i}$ ) to different headings. In equation (3.1), we assumed that all presented headings shared the same weight ( $w_{i}\;$ = 1/*n*) in the ensembled heading; however, in equation (3.2), we assumed that the weights ( $w_{i}$ ) varied among the 1st, 2nd, …, *n*th (*n* = 3) presented headings. After encoding, we assigned a weight ( $w_{0}$ or $w_{0}^{\prime }$ ) to the ensemble heading to generate the final estimate.

Three key findings would support ensemble coding in heading estimation: (1) superior performance of functions (3.1)/(3.2) over function (1) would demonstrate ensemble representation; (2) better fit of function (3.2) versus (3.1) would indicate differential weighting of headings in ensemble formation; and (3) statistically significant weights for individual headings would confirm their incorporation into the ensemble representation.

### Results and Summary

**Figure 3.**
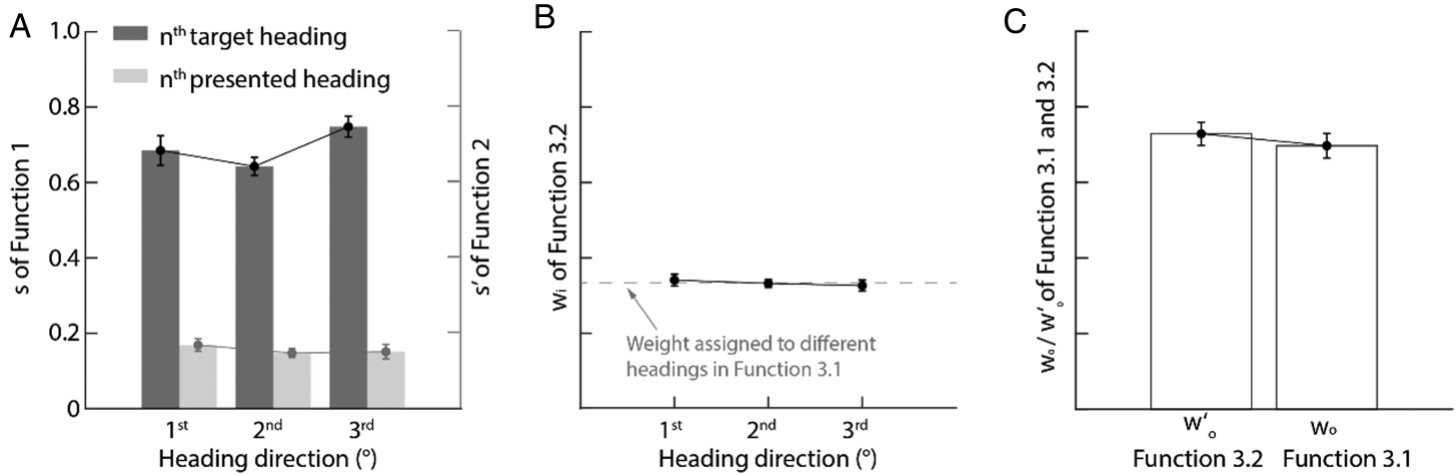
Experiment 1 results. (A) Direction-tuning slopes ( $s_{i}$ and $s_{i}^{\prime }$ ; functions 1–2) with standard error. (B) Heading-weight analysis: solid circles show mean weights (function 3.2) standard error versus theoretical uniform distribution (gray dashed line; 1/n, n = 3 in function 3.1). (C) Intercept comparison ( $w_{0}$ and $w_{0}^{\prime }$ ; functions 3.1–3.2) with standard error.

Our analysis employed two linear regression models to assess heading discrimination accuracy: function (1) modeled estimates against target headings, while function (2) used previously presented *n*th headings. As shown in Figure 3A, the significantly steeper slopes for target headings ( $s_{i}$ , dark gray) versus previous headings ( $s_{j}^{\prime }$ , light gray) demonstrated precise discrimination capability. This was statistically confirmed by a significant main effect of heading type (target vs. previous) in a repeated-measures analysis of variance (ANOVA) (*F*(1.00, 17.00) = 674.52, *p* < .001, *η*^2^ = 0.98, Greenhouse-Geisser corrected), establishing participants’ accurate discrimination of target heading directions.

In addition, as shown in Figure 3A, the slope $s_{i}$ s (dark gray bars) are also smaller than 1 (one sample t-test: *t*s (17) < −8.00, *p*s < .001, Cohen's ds > 1.89), suggesting a center bias in the heading estimation (e.g., Sun et al., 2024b).

Moreover, the slope analysis revealed a distinct serial position effect: the third target heading showed the steepest slope ( $s_{i}$ ), followed by the first, with the second heading exhibiting the shallowest slope. A repeated-measures ANOVA confirmed significant differences among target headings (*F*(1.30, 22.15) = 5.76, *p* = .018, *η*^2^ = 0.25, Greenhouse-Geisser corrected). Post hoc tests with Bonferroni correction indicated the third heading's slope was significantly steeper than both the first and second (*p*s < .041), while the first remained significantly steeper than the second (*p* = .022). This pattern demonstrates both primacy (enhanced memory for initial items) and recency (superior recall of final items) effects in heading estimation, consistent with classic memory phenomena (Anderson & Barrios, 1961; Broadbent & Broadbent, 1981).

Next, to investigate ensemble encoding, we analyzed the weighting ( $w_{i}$ ) of individual headings in the ensemble representation (functions [3.1] and [3.2]). Figure 3B reveals that all $w_{i}$ values (solid dots) were statistically indistinguishable from equal weighting (1/3, gray dashed line; one-sample t-tests: −1 < *t*s (17) < 1, *p*s > .62, Cohen's ds < 0.012), indicating equivalent contributions from each heading to the ensemble. That is, $w_{i}$ in equations (3.1) and (3.2) can be set to be fixed (1/3). Moreover, comparison of ensemble weights ( $w_{0}$ or $w_{0}^{\prime }$ ) between functions (3.1) and (3.2) showed no significant difference (*t* (17) = 1.60, *p* = .13, Hedges' *g* = 0.23; Figure 3C),^1^ suggesting that the $w_{0}$ or $w_{0}^{\prime }$ were not modulated by the weights assigned to the three headings.

Importantly, further comparisons revealed a complex pattern: while ensemble weights ( $w_{0}^{\prime }$ ) showed no difference from the slopes ( $s_{i}$ ) of the first and third target headings (−1.28 < *t*s (17) < 1, *p*s > .21, Hedges' *g*s < 0.28), the function (3.1) ensemble weight ( $w_{0}$ ) was significantly smaller than the third heading's slope ((*t* (17) = −2.20, *p* = .042, Hedges' *g* = 0.38). These findings suggest that participants likely employed ensemble encoding, and equation (3)’s explanatory power for heading estimation is close to that of equation (1). However, the clear presence of primacy (Anderson & Barrios, 1961) and recency (Broadbent & Broadbent, 1981) effects complicates definitive interpretation.

## Experiment 2

The results of experiment 1 are open to one question: whether participants reported the first/last heading or an ensemble heading. To address this concern, in experiment 2, we recruited 18 participants to complete two blocks of trials. One block comprised trials with five optic flow patterns, while the other block consisted of trials with seven optic flow patterns. Previous studies in Visual Working Memory indicates that the memory capacity is limited (Baddeley, 2012), which motivated us to compare the performance with a wider range of item numbers. Here, the heading directions were randomly selected from seven possible angles (0°, ± 10°, ± 20°, ± 30°), allowing for the repetition of the same heading within a single trial. Note that, a block design, as opposed to a randomized presentation, was employed to mitigate participant fatigue. All other parameters, procedures, and methods remained consistent with experiment 1.

**Figure 4.**
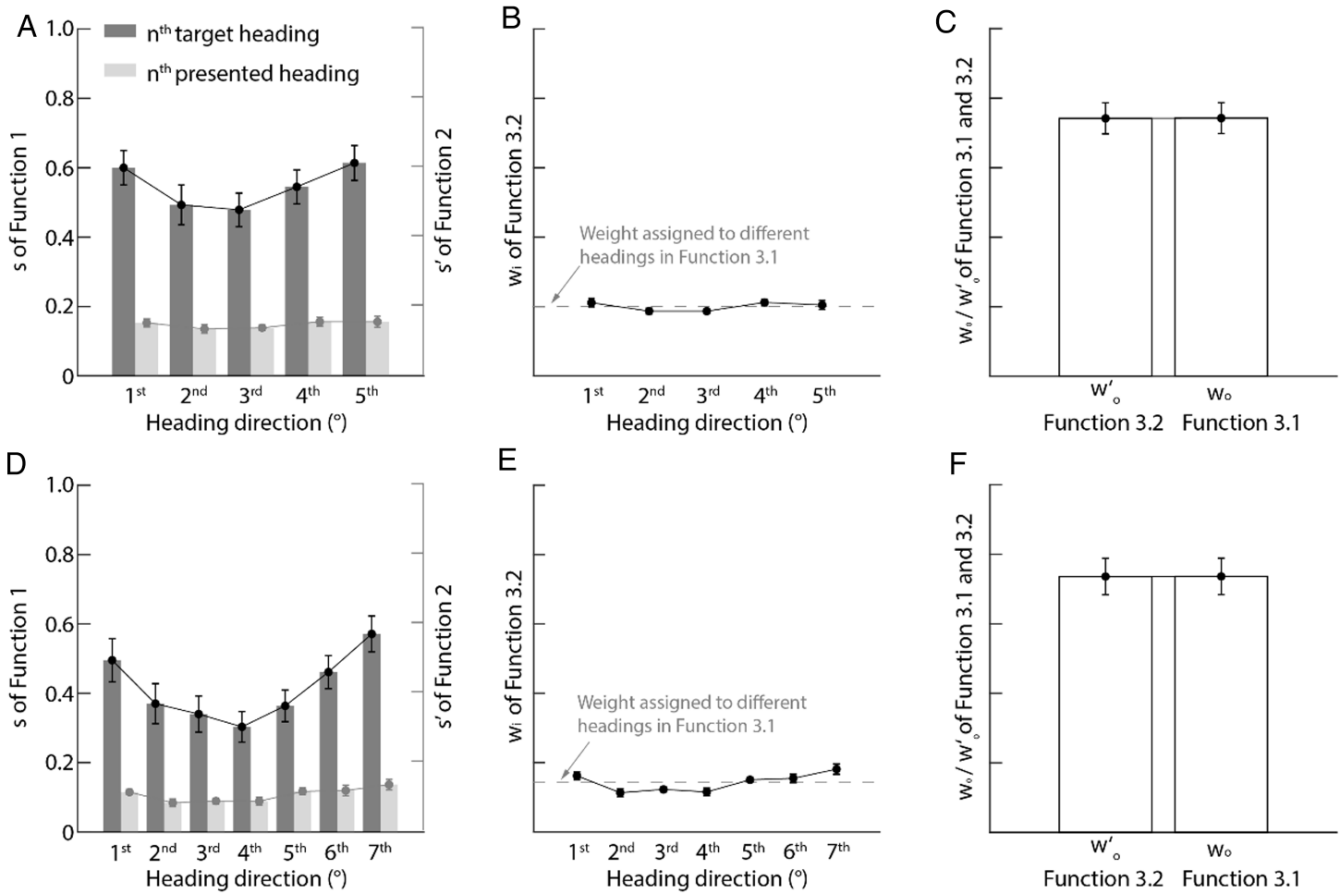
Experiment 2 results. (A) Direction-dependent slopes ( $s_{i}$ and $s_{i}^{\prime }$ in functions 1–2) with standard error. (B) Heading weights in function 3: solid dots show mean weights (function 3.2) with standard error; gray dashed line indicates uniform weights (1/n; n = 5/7) in function 3.1. (C) Comparison of intercept terms ( $w_{0}$ and $w_{0}^{\prime }$ ) between functions 3.1–3.2 with standard error.

Figure 4A and D plots the slopes ( $s_{i}$ and $s_{j}^{\prime }$ ) against different headings, showing the same pattern as in experiment 1. Firstly, the slope $s_{i}$ s (dark gray bars) are significantly larger than the slopes $s_{j}^{\prime }$ s (light gray bars), suggesting that participants can accurately discriminate the target heading directions. Secondly, the slope $s_{i}$ s (dark gray bars) are also smaller than 1, suggesting a center bias in the heading estimation of the current experiment. Thirdly, the slope $s_{i}$ of the last (fifth or seventh) target heading tends to be largest, followed by the first target heading; and the slope $s_{i}s$ of the middle (third or fourth) target heading tend to be the smallest, indicating a primacy effect (Anderson & Barrios, 1961) and recency effect (Broadbent & Broadbent, 1981) in the heading estimation of the current experiment. Furthermore, it can be also observed that the $s_{i}$ s in the three flow condition (experiment 1, Figure 3A) tend to be larger than those in the five flow condition (Figure 4A), the latter is also larger than that in the seven flow condition (Figure 4D). A repeated measures ANOVA with the flow numbers (3 vs. 5) as the between-subject factor and the heading index (first, middle, and last) as the within-subject factor showed that the main effect of the flow numbers was significant (*F*(1, 34) = 6.46, *p* = .016, *η*^2^ = 0.16); a repeated measures ANOVA with the flow numbers (5 vs. 7) and the heading index (first, middle, and last) as the within-subject factors showed that the main effect of the flow numbers was also significant (Greenhouse-Geisser corrected: *F*(1.00, 17.00) = 19.02, *p* < .001, *η*^2^ = 0.53). This suggests that the accuracy of the heading estimation decreases with the increase of the number of flow stimuli, indicating that working memory affects heading estimation from optic flow. This finding further supported the conclusion of Sun et al. (2023).

Meanwhile, the weight patterns ( $w_{i}$ s, $w_{0}^{\prime }$ s, and $w_{0}$ s; Figure 4B, C, E, and F) closely replicated the findings of experiment 1 (Figure 3B–C): (1) all $w_{i}$ approximated 1/*n* (gray dashed lines) and (2) $w_{0}$ ≈ $w_{0}^{\prime }$ across flow conditions. Crucially, unlike experiment 1, both $w_{0}$ and $w_{0}^{\prime }$ significantly exceeded the slope (*s_i_*) of the final heading (fifth/seventh) (paired t-tests: *t*s (17) > 5.43, *p*s < .001, Hedges' *g* = .61), indicating participants’ estimates reflected an ensemble average of all headings.

Table 1 compares the goodness-of-fit metrics (Deviance, AIC-Akaike Information Criterion, BIC-Bayesian Information Criterion, *R*^2^) for functions (3.1) and (3.2) across experiments. The nearly identical performance of both functions indicates that additional parameters in function (3.2) did not significantly improve variance explanation. This supports a uniform weighting strategy ( $w_{i}$ = 1/*n*; *n* = 3/5/7) in ensemble heading computation.

**Table 1.** Results of functions 3.1 and 3.2 in Experiments 1 and 2.

			Function 3.1	Function 3.2
Experiment 1	Three flow	Deviances	2258 ± 13.50	2256 ± 13.62
		AIC	2268 ± 13.0	2266 ± 13.62
		BIC	2286 ± 13.50	2284 ± 13.62
		R^2^	0.13 ± 0.013	0.13 ± 0.013
Experiment 2	Five flow	Deviances	4166 ± 76.58	4159 ± 76.95
		AIC	4780 ± 76.58	4173 ± 76.95
		BIC	4210 ± 76.58	4203 ± 76.95
		R^2^	0.19 ± 0.012	0.21 ± 0.013
	Seven flow	Deviances	5867 ± 90.71	5849 ± 92.23
		AIC	5885 ± 90.71	5867 ± 92.23
		BIC	5927 ± 90.71	5918 ± 92.23
		R^2^	0.14 ± 0.022	0.16 ± 0.025

The numbers in each cell indicate the mean index averaged across all participants and the corresponding standard error.

Moreover, an independent samples t-test showed that $w_{0}$ of the three flow condition was not significantly different from that of the five and seven flow conditions (−1 < *t*s (17) < 0, *p*s > .41, Hedges’ *g* < 0.27); and a paired samples t-test also showed that the difference in $w_{0}$ was not significantly different between the five and seven flow conditions (*t* (17) = 0.16, *p* = .88, Hedges’ *g* = 0.028). These suggest that the accuracy of the ensemble coding is not affected by the size of working memory load, indicating that the working memory does not affect the ensemble coding of heading estimation. This supports the notion that the ensemble coding can be a capacity-free process (Alvarez, 2011; Epstein & Emmanouil, 2017; Whitney & Leib, 2018).

## General Discussion

Two experiments examined the existence of ensemble coding in optic flow heading estimation. Results revealed both primacy (Anderson & Barrios, 1961) and recency effects (Broadbent & Broadbent, 1981), with estimates most closely matching the average heading sequence, which may suggest ensemble encoding in heading estimation from optic flow. This indicates a bias toward ensemble averages when recalling specific headings.

This study may provide the first empirical evidence for ensemble coding in optic-flow heading estimation. Departing from static single-flow paradigms (Crowell & Banks, 1993; Sun et al., 2023, 2024a; Warren et al., 1988), our dynamic sequential design reveals observers equally integrate multiple headings into ensemble representations (models 3.1–3.2). Critically, this suggests recalled headings reflect averaged rather than instantaneous directions—a fundamental navigation mechanism.

Meanwhile, the findings also hinted the existence of implicit ensemble coding in heading estimation. Despite explicit instructions to recall individual headings (no averaging required), estimates were consistently driven by the ensemble mean. This task-behavior dissociation reveals automatic computation of summary statistics, even when task-irrelevant, supporting ensemble coding as a fundamental perceptual mechanism (Alvarez, 2011; Haberman et al., 2009; Whitney & Leib, 2018).

Additionally, ensemble coding accuracy remained stable across varying numbers of flow patterns, supporting the notion of its cognitive load independence (i.e., capacity-free; Alvarez, 2011; Epstein & Emmanouil, 2017; Fitousi, 2025). Both experiments confirmed that grand averaging—a proxy for ensemble representation—faithfully captured individual optic flows. This aligns with domain-general ensemble coding robustness (e.g., Haberman & Whitney, 2009), including Attarha et al.'s (2014) demonstration of capacity-unconstrained coding. Even under information overload (5 vs. 7 flows), performance was comparable, reinforcing ensemble coding's automatic, capacity-free nature—a consensus across studies (Alvarez, 2011; Whitney & Leib, 2018).

These results contrast with working memory's role in individual heading estimation. As optic flow patterns increased, we observed declining accuracy for individual headings—consistent with working memory limitations. While Sun et al. (2023) indirectly implicated working memory through Electroencephalogram decoding of heading representations, our paradigm directly engaged working memory by requiring explicit recall of multiple headings. This behavioral finding partly supports Sun et al.'s (2023) neural findings and, crucially, suggests optic flow heading estimation as a cognitive process (Sun et al., 2023, 2024a) rather than purely information-driven computation (Royden & Hildreth, 1999).

Moreover, the current study indicates that ensemble coding coexists with individual heading estimation, though biasing individual representations (Brady & Alvarez, 2011; Corbett, 2017; Utochkin & Brady, 2020)—indicating preserved (but influenced) feature processing. This contrasts with studies showing exclusive ensemble perception (e.g., emotion judgments; Alvarez, 2011; Haberman & Whitney, 2007; Whitney & Leib, 2018), highlighting a stimulus-dependent duality in visual processing. Future work should examine key moderators (e.g., stimulus dynamics and task demands) to resolve this theoretical divergence.

It is important to note that the ensemble-coding interpretation was derived based on a “winner-takes-all” assumption: specifically, that either $w_{0}^{\prime }$ or $w_{0}$ are significantly larger than $s_{i}$ (experiment 2, Figure 4). However, we cannot entirely exclude alternative sequential or statistical influences, such as recency and primacy effects (Anderson & Barrios, 1961; Broadbent & Broadbent, 1981) or central tendency effects (a form of memory compression; Jazayeri & Shadlen, 2010; Olkkonen et al., 2014). Nevertheless, the current findings offer a plausible account for how observers average sequentially presented heading directions. Future studies could further dissect the contributions of these competing mechanisms.

Our current findings not only advance theoretical understanding in cognitive neuroscience but also carry practical implications such as traffic accident investigations. Our findings reflected that the visual system relies on a temporally averaged representation (integrating previous perceptual history) rather than instantaneous recording for a complex motion scenario. The current study, together with myriads of previous studies (Alvarez, 2011; Epstein & Emmanouil, 2017; Fitousi, 2025), suggested that such ensemble coding is almost automatic and capacity-unconstrainted. Thus, these findings suggesting that witness statements about travel directions may inherently incorporate this systematic bias without conscious recognition (Figure 1). Consequently, traffic authorities should account for this physiological constraint by applying temporal calibration to directional reports and maintaining appropriate flexibility in evidence evaluation during liability determination, thereby enhancing both the scientific validity and fairness of accident assessments.

In summary, this study shows that optic-flow heading estimation involves capacity-unconstrained ensemble coding operating automatically and implicitly. Crucially, while individual heading estimation remains limited by perceptual/cognitive constraints, ensemble coding better reflects natural navigation behaviors. As the first to integrate optic-flow processing with ensemble coding theory, this work provides a framework for investigating multisensory heading integration (visual/vestibular/proprioceptive) in ecological contexts.

## Notes

1. Here, we used quotation marks to highlight the ensemble heading because we were not sure that whether participants conducted ensemble encoding only with the t-test.

## References

[bibr1-20416695251377199] Alvarez G. A. (2011). Representing multiple objects as an ensemble enhances visual cognition. Trends in Cognitive Sciences, 15(3), 122–131. 10.1016/j.tics.2011.01.003 21292539

[bibr2-20416695251377199] Alvarez G. A. Franconeri S. L. (2007). How many objects can you track? Evidence for a resource-limited attentive tracking mechanism. Journal of Vision, 7(13), 14. 10.1167/7.13.14 17997642

[bibr3-20416695251377199] Anderson N. H. Barrios A. A. (1961). Primacy effects in personality impression formation. Journal of Abnormal and Social Psychology, 63, 346–350. 10.1037/h0046719 13861274

[bibr4-20416695251377199] Angelaki D. E. Gu Y. DeAngelis G. C. (2009). Multisensory integration: Psychophysics, neurophysiology, and computation. Current Opinion in Neurobiology, 19(4), 452–458. 10.1016/j.conb.2009.06.008 19616425 PMC2749464

[bibr5-20416695251377199] Attarha M. Moore C. M. Vecera S. P. (2014). Summary statistics of size: Fixed processing capacity for multiple ensembles but unlimited processing capacity for single ensembles. Journal of Experimental Psychology: Human Perception and Performance, 40(4), 1440. 10.1037/a0036206 24730736 PMC7017936

[bibr6-20416695251377199] Attneave F. (1954). Some informational aspects of visual perception. Psychological Review, 61(3), 183–193. 10.1037/h0054663 13167245

[bibr01-20416695251377199] Baddeley, A. (2012). Working memory: Theories, models, and controversies. *Annual Review of Psychology*, *63*(1), 1–29. 10.1146/annurev-psych-120710-100422 21961947

[bibr7-20416695251377199] Brady T. F. Alvarez G. A. (2011). Hierarchical encoding in visual working memory: Ensemble statistics bias memory for individual items. Psychological Science, 22(3), 384–392. 10.1177/09567976103979 21296808

[bibr8-20416695251377199] Broadbent D. E. Broadbent M. H. (1981). Recency effects in visual memory. The Quarterly Journal of Experimental Psychology Section A, 33(1), 1–15. 10.1080/14640748108400

[bibr9-20416695251377199] Burlingham C. S. Heeger D. J. (2020). Heading perception depends on time-varying evolution of optic flow. Proceedings of the National Academy of Sciences of the United States of America, 117(52), 33161–33169. 10.1073/pnas.2022984117 33328275 PMC7776640

[bibr10-20416695251377199] Chen X. DeAngelis G. C. Angelaki D. E. (2013). Eye-centered representation of optic flow tuning in the ventral intraparietal area. The Journal of Neuroscience, 33(47), 18574–18582. 10.1523/JNEUROSCI.2837-13.2013 24259579 PMC3834060

[bibr11-20416695251377199] Corbett J. E. (2017). The whole warps the sum of its parts: Gestalt-defined-group mean size biases memory for individual objects. Psychological Science, 28(1), 12–22. 10.1177/0956797616671524 27879322

[bibr12-20416695251377199] Crowell J. A. Banks M. S. (1993). Perceiving heading with different retinal regions and types of optic flow. Perception & Psychophysics, 53(3), 325–337. 10.3758/bf03205187 8483696

[bibr13-20416695251377199] D'Avossa G. Kersten D. (1996). Evidence in human subjects for independent coding of azimuth and elevation for direction of heading from optic flow. Vision Research, 36(18), 2915–2924. 10.1016/0042-6989(96)00010-7 8917793

[bibr14-20416695251377199] Epstein M. L. Emmanouil T. A. (2017). Ensemble coding remains accurate under object and spatial visual working memory load. Attention, Perception, & Psychophysics, 79, 2088–2097. 10.3758/s13414-017-1353-2 28600677

[bibr15-20416695251377199] Fetsch C. R. Deangelis G. C. Angelaki D. E. (2010). Visual-vestibular cue integration for heading perception: Applications of optimal cue integration theory. The European Journal of Neuroscience, 31(10), 1721–1729. 10.1111/j.1460-9568.2010.07207.x 20584175 PMC3108057

[bibr16-20416695251377199] Fitousi D. (2025). Capacity and architecture of emotional face-ensemble coding. Journal of Vision, 25(6), 10. 10.1167/jov.25.6.10 PMC12124146 40423625

[bibr17-20416695251377199] Gibson J. J. (1950). The perception of visual surfaces. The American Journal of Psychology, 63(3), 367–384. 10.2307/1418003 15432778

[bibr18-20416695251377199] Haberman J. Harp T. Whitney D. (2009). Averaging facial expression over time. Journal of Vision, 9(11), 1. 10.1167/9.11.1 20053064 PMC2857387

[bibr02-20416695251377199] Haberman, J., & Whitney, D. (2007). Rapid extraction of mean emotion and gender from sets of faces. *Current Biology*, *17*(17), R751–R753. 10.1016/j.cub.2007.06.039 PMC3849410 17803921

[bibr19-20416695251377199] Haberman J. Whitney D. (2009). Seeing the mean: Ensemble coding for sets of faces. Journal of Experimental Psychology. Human Perception and Performance, 35(3), 718–734. 10.1037/a0013899 19485687 PMC2696629

[bibr20-20416695251377199] Huang L. (2015). Statistical properties demand as much attention as object features. PLoS ONE, 10(8), e0131191. 10.1371/journal.pone.0131191 PMC4546565 26295808

[bibr21-20416695251377199] Jazayeri M. Shadlen M. N. (2010). Temporal context calibrates interval timing. Nature Neuroscience, 13(8), 1020–1026. 10.1038/nn.2590 20581842 PMC2916084

[bibr22-20416695251377199] Khayat N. Hochstein S. (2018). Perceiving set mean and range: Automaticity and precision. Journal of Vision, 18(9), 23, 1–14, 10.1167/18.9.23 30267075

[bibr23-20416695251377199] Layton O. W. Fajen B. R. (2016). The temporal dynamics of heading perception in the presence of moving objects. Journal of Neurophysiology, 115(1), 286–300. 10.1152/jn.00866.2015 26510765 PMC4760467

[bibr24-20416695251377199] Maus N. Layton O. W. (2022). Estimating heading from optic flow: Comparing deep learning network and human performance. Neural Network, 154, 383–396. 10.1016/j.neunet.2022.07.007 35944368

[bibr25-20416695251377199] Oberauer K. Farrell S. Jarrold C. Lewandowsky S. (2016). What limits working memory capacity? Psychological Bulletin, 142(7), 758–799. 10.1037/bul0000046 26950009

[bibr26-20416695251377199] Olkkonen M. McCarthy P. F. Allred S. R. (2014). The central tendency bias in color perception: Effects of internal and external noise. Journal of Vision, 14(11), 5. 10.1167/14.11.5 25194017

[bibr27-20416695251377199] Royden C. S. Hildreth E. C. (1999). Differential effects of shared attention on perception of heading and 3-D object motion. Perception & Psychophysics, 61(1), 120–133. 10.3758/bf03211953 10070204

[bibr28-20416695251377199] Schindler A. Bartels A. (2018). Integration of visual and non-visual self-motion cues during voluntary head movements in the human brain. NeuroImage, 172, 597–607. 10.1016/j.neuroimage.2018.02.006 29427850

[bibr29-20416695251377199] Sun Q. Wang J. Y. Gong X. M. (2024a). Conflicts between short- and long-term experiences affect visual perception through modulating sensory or motor response systems: Evidence from Bayesian inference models. Cognition, 246, 105768. 10.1016/j.cognition.2024.105768 38479091

[bibr30-20416695251377199] Sun Q. Zhan L. Z. You F. H. Dong X. F. (2024b). Attention affects the perception of self-motion direction from optic flow. iScience, 27(4), 109373. 10.1016/j.isci.2024.109373 38500831 PMC10946324

[bibr31-20416695251377199] Sun Q. Zhan L. Z. Zhang B. Y. Jia S. Gong X. M. (2023). Heading perception from optic flow occurs at both perceptual representation and working memory stages with EEG evidence. Vision Research, 208, 108235. 10.1016/j.visres.2023.108235 37094419

[bibr32-20416695251377199] Sweeny T. D. Haroz S. Whitney D. (2013). Perceiving group behavior: Sensitive ensemble coding mechanisms for biological motion of human crowds. Journal of Experimental Psychology. Human Perception and Performance, 39(2), 329–337. 10.1037/a0028712 22708744

[bibr33-20416695251377199] Utochkin I. S. Brady T. F. (2020). Independent storage of different features of real-world objects in long-term memory. Journal of Experimental Psychology: General, 149(3), 530. 10.1037/xge0000664 31414858

[bibr34-20416695251377199] Utochkin I. S. Choi J. Chong S. C. (2024). A population response model of ensemble perception. Psychological Review, 131(1), 36–57. 10.1037/rev0000426 37011150

[bibr35-20416695251377199] Warren W. H. Jr. Hannon D. J. (1988). Direction of self-motion is perceived from optical flow. Nature, 336(6195), 162–163. 10.1038/336162a0

[bibr36-20416695251377199] Warren W. H. Jr. Morris M. W. Kalish M. (1988). Perception of translational heading from optical flow. Journal of Experimental Psychology. Human Perception and Performance, 14(4), 646–660. 10.1037//0096-1523.14.4.646 2974874

[bibr37-20416695251377199] Whitney D. Leib A. (2018). Ensemble perception. Annual Review of Psychology, 69, 105–129. 10.1146/annurev-psych-010416-044232 28892638

[bibr38-20416695251377199] Xu L. H. Sun Q. Zhang B. Li X. (2022). Attractive serial dependence in heading perception from optic flow occurs at the perceptual and postperceptual stages. Journal of Vision, 22(12), 11. 10.1167/jov.22.12.11 PMC9652722 36350629

[bibr39-20416695251377199] Ying H. Burns J E. J. Choo A. M. Xu H. (2020). Temporal and spatial ensemble statistics are formed by distinct mechanisms. Cognition, 195, 104128. 10.1016/j.cognition.2019.104128 31731114

